# MINILAPAROSCOPIC APPENDECTOMY

**DOI:** 10.1590/0102-6720201600010014

**Published:** 2016

**Authors:** Lúcio Américo Della COLETTA, Bruno Ziade GIL, Renato Morato ZANATTO

**Affiliations:** Lins Santa Casa de Misericórdia Hospital, Lins, SP, Brazil

**Keywords:** Appendectomy, Minimally invasive surgery, Appendicitis

## Abstract

***Background*:**

Minilaparoscopy is considered one of the minimally invasive options available for acute appendicitis treatment, although not always employed in less complexity public health services.

***Aim*:**

Report surgical outcomes of minilaparoscopy use in acute appendicitis treatment.

**Method:**

The study included 21 patients undergoing minilaparoscopic appendectomy with instrumental of 3 mm. The following variables were analyzed: sex, age, body mass index, stage of appendicitis, surgical time, hospital stay, surgical complications, conversion rate to conventional laparoscopy or laparotomy, pain after surgery and aesthetic result.

***Results*:**

Twelve men and nine women underwent minilaparoscopic appendectomy. The average age was 27,8 years, the mean BMI was 24,8 kg/m^2^. The operative time ranged from 33 to 160 min and the average of hospital stay was three days. Among the 21 patients, 20 reported mild pain or no pain in the first postoperative day. The aesthetic result was considered "satisfactory" and "very satisfactory" by 95% of the patients.

***Conclusions*:**

The minilaparoscopy is viable technique for treating acute appendicitis with a satisfactory recovery. It combines the benefits of minimally invasive procedures with results similar to conventional techniques.

## Abstract (pt)

***Racional*:**

A minilaparoscopia é um dos métodos considerados minimamente invasivos disponíveis para o tratamento da apendicite aguda; porém, nem sempre empregada em serviços públicos de saúde de menor complexidade.

***Objetivo*:**

Relatar os resultados cirúrgicos do uso da minilaparoscopia para tratamento da apendicite aguda.

Estudo com 21 pacientes submetidos à apendicectomia por minilaparoscopia com instrumental de 3 mm. Analisaram-se as seguintes variáveis: sexo, idade, índice de massa corporal, fase da apendicite, tempo cirúrgico, período de permanência hospitalar, complicações cirúrgicas, taxa de conversão para laparoscopia convencional ou laparotomia, dor em pós-operatório e resultado estético.

Doze homens e nove mulheres foram submetidos à apendicectomia por minilaparoscopia. A média de idade foi de 27,8 anos e o IMC médio de 24,8 kg/m^2^. O tempo operatório variou de 33-160 min, com período médio de três dias de internação. Dos 21 pacientes, 20 relataram dor de leve intensidade ou nenhuma dor no primeiro dia de pós-operatório. O resultado estético foi considerado "satisfatório" e "muito satisfatório" por 95% dos pacientes.

***Conclusões*:**

A minilaparoscopia é técnica viável para tratar apendicite aguda com recuperação satisfatória. Ela agrega os benefícios dos procedimentos minimamente invasivos com resultados semelhantes às técnicas convencionais.

## INTRODUCTION

The laparoscopic technique has undergone many improvements in recent years. In an attempt to make less and less invasive procedures resulting in shorter recovery time, shorter hospital stay and fewer complications, new techniques derived from conventional laparoscopy gained notoriety in the last two decades^1^. In this context, minilaparoscopy is an option, which began in the 1990s, but soon fell into disuse because of the technical complexity, high cost of materials, little instrumental resistance, and low image quality with lower diameter of optical systems^1^^,^ 2 ^,^ 4 ^,^ 6.

In turn, in recent years it returned to gain ground in the search for procedures that could have smaller portal diameters, with less complexity and cost, when compared to other techniques considered minimally invasive as NOTES and single port^3^^,^ 4.

Not always the minilaparoscopy has been employed in medium complexity hospitals, mainly in the Brazilian Unified Health System (free of medical/hospitalization charges). The normal assistance consists in performing open appendectomy for most cases and in fewer cases conventional laparoscopy. 

In an attempt to improve the aesthetic result, decrease surgical hospital stay and promoting less pain in the postoperative period, minilaparoscopy as a technique for the treatment of acute appendicitis began the be used.

The objective of this study was to report the results of the use of minilaparoscopic technique for the treatment of acute appendicitis.

## METHOD

The study was approved by the Ethics Committee of the institution.

Twenty-one patients diagnosed with acute appendicitis were operated from February 2013 to May 2015 at the Surgery Service of the Santa Casa de Misericórdia de Lins, Lins, SP, Brazil. The following variables were analyzed: gender, age, body mass index (BMI), stage of appendicitis, surgical time, hospital stay, surgical complications, conversion rate to conventional laparoscopy or laparotomy, pain in the postoperative period and aesthetic result. The phase or degree of appendicitis was stratified according to the laparoscopic classification^5^, with grade 0=normal appendix; grade 1=hyperemia and edema; grade 2=fibrinous exudate; grade 3=necrosis; grade 4A=presence of abscess; 4B=localized peritonitis; 4C=appendiceal base necrosis; and grade 5=diffuse peritonitis.

Pain was recorded on the first day after surgery and used the visual analog pain scale classificating: no pain, mild pain, moderate pain and severe pain.

In outpatient care, usually on the 7th and 30th days after surgery, the patients were asked about their satisfaction with the cosmetic outcome of the procedure, and asked if they were "very satisfied," "satisfied," "somewhat satisfied" or " dissatisfied".

### Surgical technique

**FIGURE 1:**
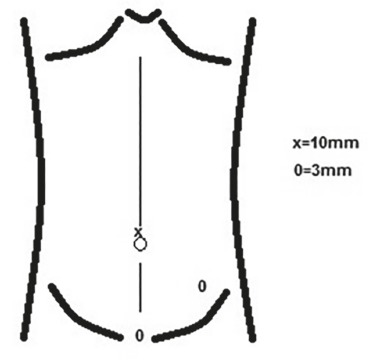
- Schematic position of 3 and 10 mm portals

The surgical technique was minilaparoscopy with three access ports, one 10 mm and two 3 mm in all patients. Under general anesthesia, pneumoperitoneum was performed by open puncture under direct vision through the umbilicus, 10 mm incision, through which it was introduced optical 30⁰ device. After examination of the cavity and confirmation of the disease, the other two punctures were performed, both 3 mm, one at the midclavicular line in the left iliac fossa, and the other in the hypogastric midline at pubic hair (Figure 1).

**FIGURE 2:**
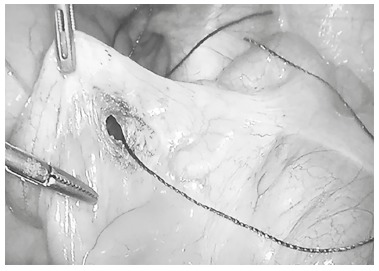
- Transfixing orifice near appendix base

**FIGURE 3:**
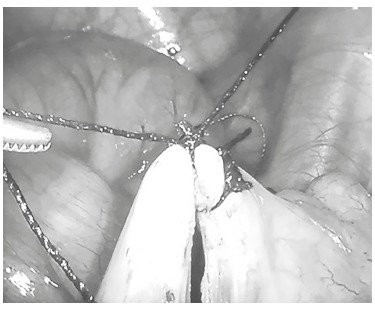
- Double ligation of the appendicular base

The appendix was identified and mobilized to make possible to dissect its mesentery in appendicular base, creating small transfixing orifice in mesoappendix (Figure 2). Through this orifice were passed two cotton stitches 2-0 and performed double endosuture ligation of appendicular base, one proximal and another distal (Figure 3).

In some cases, with appendix in retrocecal position, the right colon was mobilized by careful right parietocolic dissection in sufficient length to completely expose the appendix similar to the dissection for right colectomy. After ligation of the appendicular base, was proceeded dissection and cauterization of mesoappendix vessels with monopolar electrode in its terminal branches, near the appendix. When some large-caliber vessels were present, where electrocoagulation was not apropriated, dissection and ligation with cotton thread 2-0 were performed.

After completion of the mesoappendix dissection, the base incision with scissor 3 mm was conducted, cauterizing its stump which has not buried or invaginated.

The appendix was placed in a sterile plastic bag (adapted from sterile glove finger). Then, cleaning and irrigation of the cavity with 0.9% saline solution were done when needed, and appendix was ​​removed from the cavity through the umbilical 10 mm incision. At the end of the procedure, intradermal suturing was done only in 10 mm incision.

## RESULTS

Of the 21 patients, 12 (57.1%) were men and nine (42.9%) women with a mean age of 27.8 years. Also BMI was analyzed, ranging from 18-35 kg/m^2^, with an average value of 24.8 kg/m^2^. The shorter surgical time was 33 min and 160 min for the longer procedure, average 64.1 min. Regarding the hospitalization period, average was three days (2-7, Table 1).

**TABLE 1:** - Demographic data, duration of surgery and hospital stay

**Variables**	**Minimum**	**Maximum**	**Average**
Age years)	6	67	27.8
BMI (kg/m2)	18	35	24.8
Surgical time (min)	33	160	64.1
Hospital stay (days)	2	7	3

BMI=body mass index

As for disease staging, there was grade 2 prevalence (42.8%) and only one patient with grade 5. Pain was assessed on the first day after surgery and patients responded according to the visual analog scale pain - EVA: 11 "no pain"; 9 "slight pain", one "moderate pain" and no "intense pain". As for the aesthetic result in outpatient 95% said they were "very satisfied" or "satisfied" and there was just one "somewhat satisfied" with the patient's aesthetic appearance, due to conversion to laparotomy (Table 2).

**TABLE 2:** - Appendicitis phases and postoperative variables

**Variables**	**n**	**%**
Apendicitis stages		
Grade 0	1	4.8
Grade 1	3	14.2
Grade 2	9	42.8
Grade 3	4	19.1
Grade 4 (A, B, C)	3	14.2
Grade 5	1	4.8
Pain on day 1 postoperatively		
None	11	52.4
Light	9	42.8
Moderate	1	4.8
Intense	-	-
Aesthetic result		
Very satisfied	17	81
Satisfied	3	14.2
Somewhat satisfied	1	4.8
Dissatisfied	-	-

Only in this case there was need for conversion to laparotomy due to retrocecal appendicitis (with 10 days of evolution), which caused necrosis of the posterior wall of the cecum and ascending colon and diffuse peritonitis, being chosen right paramedian laparotomy and partial colectomy. Of the patients, the only complication after surgery (superficial wound infection) occurred in the same case needing laparotomic conversion.

## DISCUSSION

Because the diameter variations of the materials used, there is still controversy about the concept of minilaparoscopy. Accordingly Avila et al.^6^ classification, there was a proposition to standardize minimally invasive procedures as conventional minilaparoscopy (diameter 4.9 to 3.5 mm), modern minilaparoscopy (diameter of 3.4 mm to 2 mm), micro minilaparoscopy (1.9 to 0.5 mm) and ultramicro minilaparoscopy (diameter less than 0.5 mm). In contrast, some authors consider as minilaparoscopic any technique that uses materials with diameter less than or equal to 5 mm^1^^,^ 8. In turn, for Carvalho et al.^4^ the use of portal equal to 5 mm outside the umbilicus is considered as a hybrid technique or conversion to conventional laparoscopy. Even for those authors^4^ the minilaparoscopy is understood as a technique whose sum of the incisions does not exceed 20 mm. Simple modifications in the initial minilaparoscopic technique brought significant advances in the acceptance and applicability of the method, especially for performing cholecystectomies and appendectomies. The making of endossutures offers the advantage of dispensing metal clips and two optical systems of different diameters in a single procedure, with the consequent reduction in cost and timing^9^^,^ 10.

Similarly, with this technique, was dispensed the use of metal clips, clamps and laparoscopic staplers. Moreover minilaparoscopy material, that is reusable and has long durability, was used only low cost cotton threads for ligations.

It may be questioned a possible increase in surgical time due to the need of making endosutures. Sato et al.^10^ described minilaparoscopic technique using a retrator loop for appendix manipulation, and reported mean operative time of 65 min. The experience reported by Paquentín et al.^11^ shows mean operative time of 48.52 min for minilaparoscopic appendectomy; however, the study does not detail the technical steps and materials used for the procedure. The studies Croce et al.^12 ^and Di Lorenzo et al.^13^ showed similar average surgical time (35 and 34 min, respectively); however, they used a 2 mm optical device, ultrasonic shears and endoloops. Mostafa et al.^14^ reported minilaparoscopic technique with an average duration of 55 min and used two optical systems and endostaplers. In this study, the mean operative time was 64.1 min, a period that does not differ in important ways from these studies.

Another point to be considered to perform the technique is in BMI. Noack et al.^8^ 13 patients with BMI greater than 30 kg/m^2^ underwent cholecystectomy, three by minilaparoscopic technique and a significant increase in operative time and pain were related in these patients. Mostafa et al.^14^ excluded use of minilaparoscopic technique in patients with morbid obesity. Schauer et al.^1^ considered obesity and cases of complicated appendicitis as relative contraindication to perform the minilaparoscopic art. In this study, it was used in patients with BMI greater than 30 kg/m^2^, and there was no significant increase in surgical time, need for conversion, damage to material or reported complications, which can be explained, perhaps, by improving material quality using a 10 mm optics and simplified technique.

As for the aesthetic result, minilaparoscopic proved to be promising. There is scientific evidence on the satisfactory cosmetic result, which is considered one of technique attractive^1^^,^ 4 ^,^ 10 ^,^ 15.

When using material with reduced diameter providing low friction and gripper devices, they have promoted more precise and delicate movements generating less trauma to the abdominal wall, and thus favoring the healing process. Another result that can be attributed to these factors, is less pain postoperatively due to less handling and peritoneal trauma^4^^,^ 8. Considerable percentage of patients showed up "very satisfied" and "satisfied" (over 95%) with the aesthetic result, which agrees with the cosmetic results obtained by Noack et al.^8^.

Despite the difficulty of acceptance of laparoscopic techniques, especially in smaller centers, defenders refer as attractive the small size of the incisions, less surgical trauma, important aesthetic gain and possible reduction of complications related to abdominal wall^1^^,^ 2 ^,^ 4.

## CONCLUSION

The minilaparoscopy is safe and effective option for the treatment of acute appendicitis in its various stages and in different age groups, with complication rates and conversion similar to conventional laparoscopic technique, aesthetically satisfying and less postoperative pain.
